# Radiomic Phenotype of Periatrial Adipose Tissue in the Prognosis of Late Postablation Recurrence of Idiopathic Atrial Fibrillation

**DOI:** 10.17691/stm2023.15.2.05

**Published:** 2023-03-29

**Authors:** J.N. Ilyushenkova, S.I. Sazonova, E.V. Popov, R.E. Batalov, S.M. Minin, A.B. Romanov

**Affiliations:** Senior Researcher, Nuclear Medicine Department; Cardiology Research Institute, Tomsk National Research Medical Center of the Russian Academy of Sciences, 111a Kievskaya St., Tomsk, 634012, Russia;; Head of Nuclear Medicine Department; Cardiology Research Institute, Tomsk National Research Medical Center of the Russian Academy of Sciences, 111a Kievskaya St., Tomsk, 634012, Russia;; PhD Student, Nuclear Medicine Department; Cardiology Research Institute, Tomsk National Research Medical Center of the Russian Academy of Sciences, 111a Kievskaya St., Tomsk, 634012, Russia;; Leading Researcher, Department of Surgical Treatment of Advanced Heart Rhythm Disorders and Pacing; Cardiology Research Institute, Tomsk National Research Medical Center of the Russian Academy of Sciences, 111a Kievskaya St., Tomsk, 634012, Russia;; Head of the Nuclear Diagnosis Unit, Department of Radiological and Functional Diagnosis; Meshalkin National Medical Research Center of the Ministry of Health of the Russian Federation, 15 Rechkunovskaya St., 630055, Novosibirsk, Russia; Deputy Director for Science; Meshalkin National Medical Research Center of the Ministry of Health of the Russian Federation, 15 Rechkunovskaya St., 630055, Novosibirsk, Russia

**Keywords:** atrial fibrillation, radiomics, periatrial adipose tissue, catheter ablation, computed tomography, postablation recurrence

## Abstract

**The aim of the study** is to find new predictors of postablation atrial fibrillation (AF) recurrence in patients with lone AF using a texture analysis of the periatrial adipose tissue (PAAT) of the left atrium.

**Materials and Methods:**

Forty-three patients admitted for lone AF catheter ablation, who had undergone multispiral coronary angiography, were enrolled in the study. PAAT segmentation was performed using 3D Slicer application followed by extraction of 93 radiomic features. At the end of the follow-up period, patients were divided into 2 groups depending on the presence or absence of AF recurrence.

**Results:**

12 months of follow-up after catheter ablation, postablation AF recurrence was reported in 19 out of 43 patients. Of 93 extracted radiomic features of PAAT, statistically significant differences were observed for 3 features of the Gray Level Size Zone matrix. At the same time, only one radiomic feature of PAAT, Size Zone Non Uniformity Normalized, was an independent predictor of postablative recurrence of AF after catheter ablation and 12 months of follow-up (McFadden’s R^2^=0.451, OR — 0.506, 95% CI: 0.331‒0.776, p<0.001).

**Conclusion:**

The radiomic analysis of periatrial adipose tissue may be considered as a promising non-invasive method for predicting adverse outcomes of the catheter treatment, which opens the possibilities for planning and correction of patient management tactics after intervention.

## Introduction

Catheter ablation is one of the most effective and safe method of treating atrial fibrillation (AF).

At the same time, AF recurrence rate after a single procedure reaches 50% [1]. Presently, a large number of risk factors have been identified, including those of inflammatory genesis [2] which are independent predictors of AF recurrence [3]. These factors were used to create rating scales for prediction of the results of catheter ablation and arrhythmia [4]. These scales are applicable to different cohorts of patients [3]. However, it is not always possible to establish the cause of AF development. None of the existing rating scales can be used for this cohort to assess the risks of AF recurrence after catheter ablation. This creates the need for finding new predictors of postablative recurrence.

In light of the foregoing, periatrial adipose tissue (PAAT) as a structure contacting most closely with the atrial myocardium and pulmonary vein ostia is of great interest in regard to studying not only the mechanism of arrhythmogenesis but also postablation AF recurrence [5]. The adipose tissue has been shown to be a paracrine organ capable of causing a proarrythmogenic effect mainly due to infiltration of myocardium by adipocytes and adipokine secretion promoting structural remodeling of the atrial myocardium [6, 7]. Studies based on determination of the mass, thickness, and attenuation of PAAT by computed tomography confirm their association with arrhythmia recurrence after catheter treatment [6-8].

Today, the medical imaging technologies have reached a higher level owing to the extraction of new quantitative features from standard medical images used for the detection of clinically significant information obscure to the naked eye [9, 10], opening new perspectives for personalized medicine [11]. Radiomics has found its application in the evaluation of atherosclerotic plaque structures in the coronary arteries [12, 13] and in the prediction of complications after myocardial infarction [14]. Promising are also investigations in the field of myocardial pathology (cardiomyopathy, myocarditis), coronary calcium assessment, and prediction of major adverse cardiovascular events, as well as investigations of the epicardial adipose tissue (EAT) in various cardiovascular diseases [15, 16] including AF [17].

**The aim of the study** is to find new predictors of postablation atrial fibrillation recurrence in patients with lone atrial fibrillation using a texture analysis of the periatrial adipose tissue of the left atrium.

## Materials and Methods

The study included 43 patients (35 men, 8 women) with lone AF referred to the clinic for catheter ablation. They underwent multispiral coronary angiography (MDCT-CA) which was performed according to clinical indications [18] and for preoperative evaluation of the pulmonary vein ostia dimensions, anatomy of their draining, measurement of the left atrial size and volume [19].

The following criteria were used for inclusion into the study: 18–60 years of age, paroxysmal, persistent, or long-standing persistent form of AF [4] of unknown etiology; informed consent of patients for participation in the study.

Exclusion criteria were as follows: cardiovascular system pathology including hypertension, hypercholesterinemia, coronary heart disease, infarction or stroke in the history, congestive heart failure (EF less than 50%), peripheral vessel diseases, cardiac valvulopathy, intracavitary thrombi and the effect of spontaneous contrasting shown by the data of transesophageal ultrasound examination; diabetes, thyroid gland, and urinary system pathology. Significant artifacts on CT images, inconsistency of the X-ray tube parameters (described in the “Protocol of MDCT-CA” subsection) during scanning were also criteria for the exclusion from the study.

### Characteristics of the patients

The clinical characteristics of the patients are presented in Table 1.

**Table 1:** Clinical characteristics of patients with atrial fibrillation

Description	Values
Gender, n (%):	
male	35 (81)
female	8 (19)
Age (years)*	42 [35; 47]
Duration of atrial fibrillation (years)*	4 [2; 7]
Type of atrial fibrillation, n (%):	
paraxysmal	20 (47)
persistent	12 (28)
long-standing persistent	11 (25)
Absence of atrial fibrillation recurrence during 12 months of follow-up, n (%)	24 (56)
Body mass index*	28 [24; 30]
Body mass index, n (%):	
19.0–24.9 (normal)	12 (18)
25.0–29.9 (excessive body mass)	18 (52)
30.0–34.9 (obesity I degree)	13 (30)
Glucose (mmol/l)*	4 [3; 4]
Glucose tolerance test (mmol/l)*	6 [5; 7]
Total cholesterol (g/ml)*	3.9 [3.5; 4.8]
High-density lipoproteins (mmol/l)*	1.6 [1.4; 1.9]
Low-density lipoproteins (mmol/l)*	2.4 [2.1; 2.6]
Smoking, n (%)	8 (19)
Alcohol consumption, n (%)	0
Obstructive sleep apnea syndrome, n (%)	0
Office blood pressure, n (%):	
normal	30 (70)
increased	13 (30)
Ejection fraction (%)*	66 [60; 66]
End-diastolic volume (ml)*	112 [108; 125]
End-systolic volume (ml)*	37 [37; 52]
Diameter of the left atrium (mm)*	40 [35; 44]
Maximum volume of left atrium (cm^3^)*	101 [95; 119]
CT signs of coronary artery atherosclerosis, n (%)	0

* data is presented as Me [Q1; Q3]*.*

Antiarrhythmic and antithrombotic therapy used at the time of patient inclusion into the study was administered according to the ABC strategy [3] and was not changed during the first 3 months of the follow-up. Drug therapy consisted of beta-blockers (30% of patients), amidodarone/sotalol/propafenone (93% of patients), and anticoagulants (100% of patients).

### Protocol of MDCT-CA

During the study, all patients had a sinus rhythm with a heart rate of 50–65 bpm. In patients with persistent and long-standing persistent AF, electrical cardioversion was conducted under intravenous sedation in the intensive care unit to restore the sinus rhythm before the investigation.

MDCT-CA was performed using a 64-detector CT scanner (Discovery NM/CT 570c, GE Healthcare, USA). The investigation consisted of two scanning phases. The first non-enhanced phase (Ca-scoring) was performed in the ECG-synchronized mode at 120-mA tube current, 400-ms tube rotation speed, 1.25-mm slice thickness.

The angiogrephic phase of investigations was performed in the retrospective ECG-synchronized helical scan with 120–140-kV tube voltage, 200–700-mA current (depending on patient’s body mass), 400-ms tube rotation speed, 0.625-mm slice thickness, and pitch of 0.18:1–0.24:1 (depending on HR). Contrasting of the coronary arteries, large vessels, and cardiac cavities was carried out by intravenous infusion of 70–110-ml iodine-containing contrast agent (adjusted to the body mass) at iodine concentration of 350–370 mg/ml and 5-ml/s flow rate. The data obtained were reconstructed in the diastole phase (mainly 75% of the R–R interval duration) and analyzed using Advantage Workstation 4.6 (GE Healthcare, USA) [20].

The total effective dose per patient was 4.0–5.5 mSv.

### Segmentation of the periatrial adipose tissue and extraction of radiomic features

**Figure 1.**
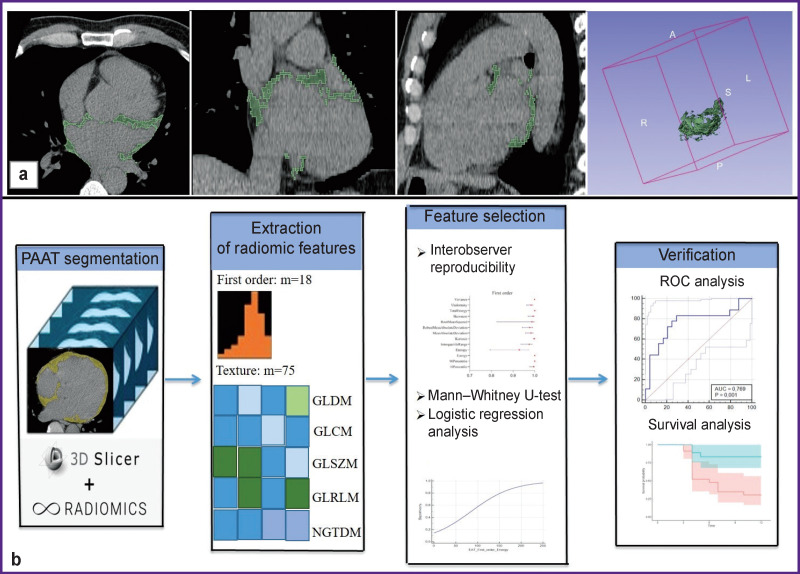
Study design for periatrial adipose tissue segmentation and extraction of radiomic features: (a) segmentation of periatrial fat on non-enhanced CT scans, from left to right: axial slice, frontal slice, sagittal slice, 3D imaging of the segmented periatrial adipose tissue; (b) schematic presentation of the study design. Designations are given in the text

Segmentation and the following radiomic PAAT analysis were performed using non-enhanced ECG-synchronized series of 3D DICOM-images (Ca-scoring series) which were exported to the 3D Slicer software application (USA) [21]. The PAAT was segmented by variable sized hand tool in the range of adipose tissue attenuation values from ‒190 HU to –30 HU slice by slice around the left atrium including the pulmonary vein ostia (Figure 1).

**Figure 2.**
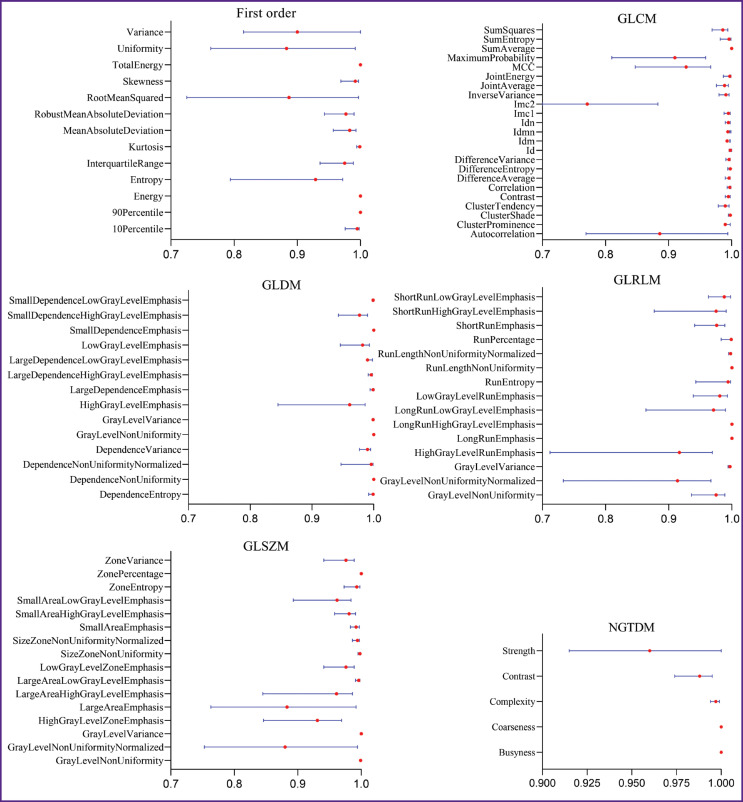
Forest plot of interobserver reproducibility (mean, 95% CI) of radiomic features grouped by matrices. Designations are given in the text

SliсerRadiomics module (v. 4.10.2) was used to calculate the values of PAAT mean attenuation, volume, and radiomic PAAT features which included 18 parameters of the first order statistics, 24 Gray Level Co-Occurrence Matrix (GLCM) features, 16 Gray Level Run Length Matrix (GLRLM) features, 14 Gray Level Dependence Matrix (GLDM) features, 16 Gray Level Size Zone Matrix (GLSZM), and 5 Neighboring Gray-Tone Difference Matrix (NGTDM) features. A full list of radiomic features is presented in Figure 2.

All investigations including interpretation of images, PAAT segmentation, and extraction of radiomic features were performed by two radiologists with over 10 years of experience in the field of thoracic and cardiovascular multimodal imaging who were unaware of the clinical history of these patients and the results of the previous analysis.

### Protocol of radiofrequency ablation of ectopic atrial fibrillation foci

The Seldinger technique was used to obtain access to the femoral vein under medicamentous sedation and local infiltration anesthesia. The right femoral vein was punctured three times to install three introducers: 9Fr, 6Fr, and Fast-Cath 8.5Fr. The NaviStar ThermoCool 3.5 mm (Biosense Webster, USA) and 4-pole diagnostic Viking catheters (Boston Scientific, USA) were advanced through the 9Fr and 6Fr introducers to the cavity of the right atrium. The interatrial septum was punctured under the control of transesophageal echocardiography using Fast-Cath 8.5Fr introducer and BRK-1 needle (St. Jude Medical, USA); thereafter, the Lasso ablation and circular electrodes (Biosense Webster, USA) were advanced to the left atrium. After interatrial septum puncture, heparin infusion was started maintaining the activated clotting time between 300 and 350 s throughout the operation. Electrophysiological investigation was conducted using Elcart hardware-software complex (Electropulse, Russia). A 3D CARTO 3 nonfluoroscopic system (Biosense Webster, USA) with the application of FAM technology was employed for anatomical reconstruction of the left atrium. The circular electrode was alternately placed in each pulmonary vein and recorded electrical activity in these veins. Radiofrequency energy was applied with radiofrequency RF100-TZ destructor (Electropulse, Russia) with 45-W power control and temperature of 500°С. The CoolFlow pump (Biosense Webster, USA) was used for irrigation at flow rate of 17 ml/min. Point-to-point formation of the radiofrequency lesion lines was implemented by continuous application of the radiofrequency energy. The time of energy application at each point was 20– 30 s until the decrease of the atrial potential. Besides, radiofrequency destruction was performed around each ostium of the pulmonary vein with a margin of 0.5–1.0 cm. The electrophysiological criterion of the left vein isolation was the disappearance of its potential at the circular Lasso electrode. The “input” and “output” block was registered during stimulation of the left vein and left atrium [22].

### The follow-up period and endpoints

Patients were under outpatient monitoring for 12 months after catheter ablation. To reveal AF recurrences, patients underwent Holter ECG monitoring 3, 6, and 12 months after the intervention. Episodes of AF rhythm lasting more than 30 s were criteria of recurrence. The first endpoint of the study was AF recurrence within the period from 3 to 12 months after ablation; the main unfavorable cardiovascular events were the second endpoint.

At the end of the follow-up period, patients were divided into two groups: group 1 — patients with AF recurrence; group 2 — patients without AF recurrence.

### Statistical data processing

Statistical processing of the data was carried out using the R (v. 4.1.3) software environment using the following packages: ggplot2, peacock.test, gMWT, WMWssp, linkspotter, ROCS, jackknifeKME, KMunicate, manhattanly, blorr, irrICC, extracted from Comprehensive R Archive Network (CRAN). The quantitative data were presented as median and quartiles — Me [Q1; Q3]; arithmetic mean ± standard deviation as M±σ; the qualitative features were presented in absolute and percentage values. Statistical significance of the intergroup differences was evaluated according to the nonparametric Mann–Whitney and Kruskal–Wallis tests. Categorical variables were compared using Fisher’s exact test. The logistic regression analysis was applied to determine significant radiomic predictors of AF recurrences and to create a prognostic model. Sensitivity and specificity values for the model were calculated based on the generally accepted formulas and ROC curves. Kaplan–Meier method was employed to measure a fraction of patients without the event occurrence (AF recurrence) at any time over the entire follow-up period. Kaplan–Meier curves were compared using a log-rank test.

Interobserver reproducibility was quantified by means of a two-way random model (with absolute agreement) of interclass correlation (ICC) coefficients and their 95% confidence interval (CI) for each feature. The ICC values were interpreted in the following way: poor (ICC CI < 0.5), moderate (0.5 < ICC CI < 0.75), good (0.75 < ICC CI < 0.9), and excellent (ICC CI > 0.9) reliability [23].

The correlation analysis was performed using Spearman’s rank correlation coefficient (ρ).

## Results

### The main CT characteristics of periatrial adipose tissue

**Figure 3.**
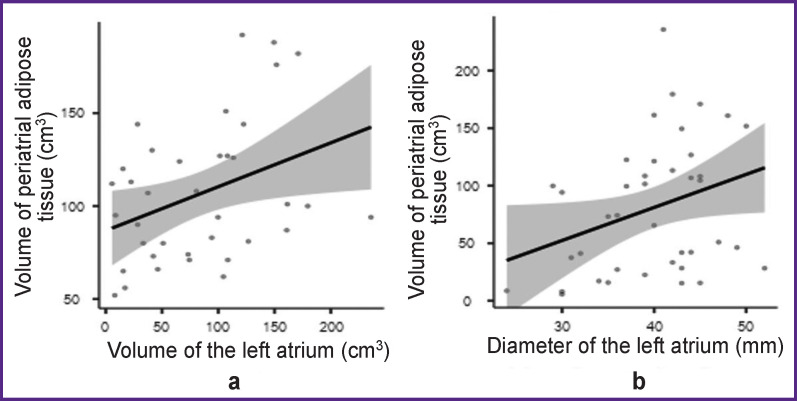
Scatterplot for studying correlation between the indicators of (a) volume of periatrial adipose tissue and left atrial volume, (b) volume of periatrial adipose tissue and left atrial diameter Confidence intervals are highlighted in gray

The PAAT median volume was 77 [63; 97] cm^3^, attenuation was –81 [–79; –81] HU (Hounsfield units). A weak positive correlation was found between the values of PAAT volume, volume and diameter of the left atrium (ρ=0.337, p=0.041 and ρ=0.352, p=0.026, respectively). A scatterplot presented in Figure 3 shows that the PAAT volume increases with the increase of the volume and diameter of the left atrium. No relationships between other clinical risk factors (AF type, arrhythmologic history, smoking, BMI) of AF development and its postablation recurrence have been detected.

### Results of the follow-up 12 months after the intervention

12 months after catheter ablation, AF recurrence was observed in 19 patients (44%). Statistically significant differences and correlations between the main risk factors of the AF development and recurrence, PAAT volume and attenuation have not been revealed (Table 2).

**Table 2:** Clinical characteristics of patients with and without postablation recurrence after 12 months of follow-up

Description	Group 1 recurrence of atrial fibrillation (n=19)	Group of 2 without recurrence atrial fibrilation (n=24)	p-value
Gender, n (%):			
men	17 (90)	18 (75)	0.11
women	2 (10)	6 (25)	0.09
Age (years)*	43.80±12.17	40.60±7.19	0.10
Duration of atrial fibrillation (years)*	5.47±4.14	4.75±3.33	0.65
Type of atrial fibrillation, n (%):			
paraxysmal	6 (32)	14 (58)	0.13
persistent	8 (42)	4 (17)	0.66
long-standing persistent	5 (26)	6 (25)	0.21
Body mass index*	27.90±2.67	28.30±3.42	0.17
Smoking, n (%)	4 (21.0)	4 (16.6)	0.80
Office blood pressure, n (%):			
normal	14 (73.7)	16 (66.7)	0.14
increased	5 (26.3)	8 (33.3)	0.09
Ejection fraction (%)*	61.70±12.25	64.50±7.36	0.61
End-diastolic volume (ml)*	120.10±37.16	112.50±17.70	0.09
End-systolic volume (ml)*	50.0±37.44	40.40±10.22	0.61
Diameter of the left atrium (mm)*	39.80±7.14	39.40±50.89	0.58
Maximum volume of the left atrium (сm^3^)*	104.20±38.70	100.0±35.24	0.20
Periatrial adipose tissue volume (сm^3^)*	86.20±51.06	83.67±61.18	0.07
Periatrial adipose tissue attenuation (HU)*	–80.0±5.13	–81.0±5.26	0.23

* data is presented as M±σ.

### Interobserver reprodicibility

Interobserver reproducibility was good and excellent for all radiomic features. ICC for the PAAT volume was 0.835 (95% CI: 0.812–0.867, p<0.001), and for the PAAT attenuation it was 0.991 (95% CI: 0.977–0.996, p<0.001). Figure 2 illustrates the interclass correlation coefficients (mean, 95% CI) for each radiomic feature grouped by matrices.

### Results of radiomic analysis of the periatrial adipose tissue

**Figure 4.**
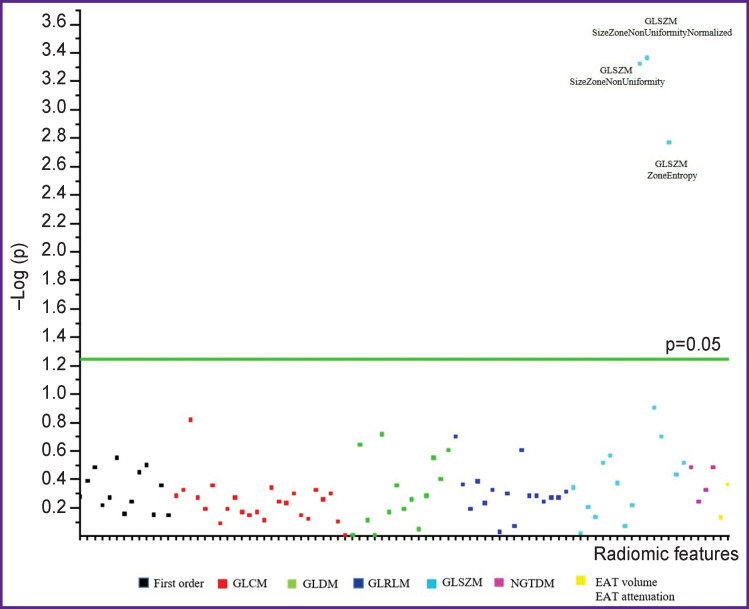
Manhattan plot of p-values for Mann–Whitney U-test comparing the base periatrial adipose tissue characteristics and all radiomic features between groups 1 and 2 Y-axis — negative log of p-values for each of 93 radiomic features built along the X-axis. Features located above the green line are statistically significant. Designations are given in the text

Of 93 extracted radiomic features, statistically significant differences were observed for three features of the GLSZ matrix: Size Zone Non Uniformity (р<0.001), Size Zone Non Uniformity Normalized (р<0.001), and Zone Entropy (р<0.001) (Figure 4). Statistically significant positive or negative correlations were noted between the diameter of the left atrium and Size Zone Non Uniformity Normalized (ρ=0.337; p=0.034), Size Zone Non Uniformity (ρ=0.350; p=0.027), Zone Entropy (ρ=0.334; p=0.03).

**Figure 5.**
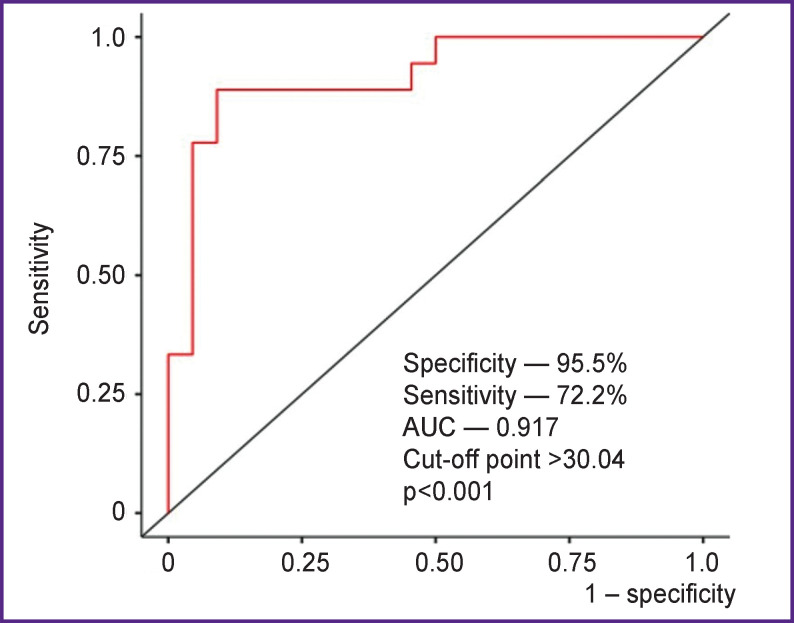
ROC curve showing diagnostic performance of the Size Zone Non Uniformity Normalized GLSZ matrix in predicting postablation recurrence of atrial fibrillation

According to the results of the logistic regression analysis, only one PAAT radiomic feature, Size Zone Non Uniformity Normalized, was an independent predictor of AF recurrence after catheter ablation during 12-month follow-up period (McFadden’s R^2^=0.451; OR — 0.506; 95% CI: 0.331–0.776; p<0.001). Based on the ROC analysis results, the logistic model demonstrates high values of sensitivity and specificity in the prediction of postablation AF (cut-off point >30.04; 95.5% specificity, 72.2% sensitivity, 85% accuracy, AUC — 0.917; p<0.001) (Figure 5). It should be noted that clinical risk factors do not possess these properties.

**Figure 6.**
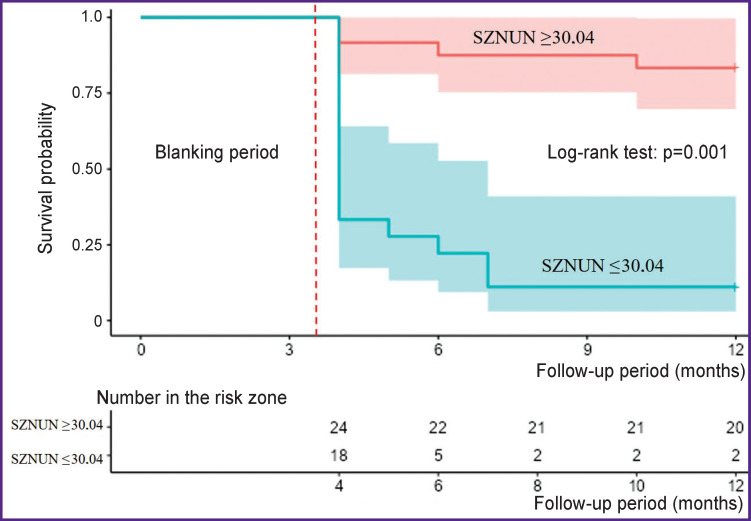
Kaplan–Meier curves estimating the recurrence-free atrial fibrillation rate SZNUN — Size Zone Non Uniformity Normalized

The Kaplan–Meier analysis has shown that the value Size Zone Non Uniformity Normalized of the GLSZ matrix over 30.04 exceeds considerably the risk of AF recurrence post ablation (Figure 6).

## Discussion

In recent years, great attention has been paid to PAAT as a structure being in direct contact with atrial myocardium and pulmonary vein ostia. Interrelations between PAAT thickness, mass, attenuation, AF development, and its severity have been established [24, 25]. From a pathophysiological point of view, periatrial fat is capable of causing a proarrhythmogenic effect by means of two mechanisms of action. The first of them, mechanostructural, occurs due to direct infiltration of myocardium by adipocytes [5]. The second mechanism, paracrine, is brought about owing to the production of proinflammatory cytokines by the adipose tissue itself and infiltration by macrophages [5, 25]. Finally, it results in electrical and structural remodeling of the left atrial myocardium, with subsequent formation of a substrate hindering adequate pulse conduction.

In relation to prediction of AF recurrence, PAAT characteristics are given less attention than EAT. Nevertheless, certain results have also been achieved in this respect. Thus, Kocyigit et al. [8] have shown that PAAT thickness according to the CT data is an independent predictor of postablation AF recurrence in patients with the preserved eject fraction of the left ventricle without valvular pathology. In their study, Ciuffo et al. [9] have demonstrated that PAAT attenuation is an independent predictor of the AF recurrence after ablation even after the correction for clinical risk factors. Based on measuring the adipose tissue attenuation in the region of the posterior wall of the left atrium, Gaibazzi et al. [26] have identified an independent association of the PAAT volume and its attenuation with AF presence and postablation recurrence. Besides, PAAT attenuation below –76 HU allowed for recurrence prediction with high sensitivity but sufficiently low specificity [26]. In the similar study by El Mahdiui et al. [6], PAAT attenuation below –96 HU was also a promising predictor of arrhythmia recurrence. The authors believe that the reduction of adipose tissue attenuation may be considered the marker of the inflammatory process in the fatty tissue surrounding the atrium or in the atrial myocardium [6, 26]. However, the authors do not provide their own evidence for their hypothesis, such as analysis of adipokines, pro-inflammatory cytokines and histological analysis. Besides, it should be noted that these studies included patients having clinical risk factors of AF development and recurrence, which could also influence the metabolic processes going on in the adipose tissue [5]. For this reason, we have focused our attention on a separate cohort of patients with atrial fibrillation, the reason of which is difficult to identify.

In our study, we did not find any correlation between the basic CT characteristics of PAAT (volume and attenuation) and the development of late AF post ablation, which is not in line with the results of El Mahdiui [6] and Gaibazzi [26]. This disagreement may be due to the different methodology of the study, as we used the entire volume of the periatrial adipose tissue. Besides, we calculated the mean value of the adipose tissue attenuation in the whole segmented volume. It cannot be ruled out that fat attenuation in different parts (posterior wall, anterior wall, pulmonary vein ostia) is heterogeneous and depends on the severity of the ongoing inflammatory process [6]. The inflammatory process in adipose tissue is closely associated with the atherosclerotic process in the coronary vessels, obesity, diabetes mellitus [27-29], which are the recognized risk factors of AF development and recurrence. Considering the fact that patients in our study were young and middle-aged, we believe that inflammatory process had a minimal impact on the postablation recurrence and mechanostructural mechanism of action becomes a key issue. This hypothesis is proved by a moderate positive correlation between the PAAT volume, volume of the left atrium and its diameter. Nevertheless, application of the texture analysis of the CT images of the atrial adipose tissue might improve the understanding of the processes going on in PAAT in regard to its effect on the AF recurrence postablation.

Successful and wide application of radiomics in clinical practice is possible only if there is good reproducibility of radiomic parameters which is achieved by using a single criterion of image recording and post-processing. Texture features are known to be sensitive to the method of segmentation (manual or automatic), X-ray tube parameters, algorithm of image reconstruction, and the type of the software used [30, 31]. For this reason, we have chosen the unenhanced series of images, performed according to the standardized Ca-scoring protocol, for the analysis [32]. This approach gives the possibility to maintain high reproducibility of the results and to carry out multicenter investigations. Usage of the contrast-enhanced series for extraction of radiomic features has the advantage only in a higher image resolution, since the X-ray tube parameters are adjusted individually depending on body mass, HR, and the amount of the contrast substance, which inevitably influences the radiomic features. Tests for reproducibility of the volume, attenuation, and PAAT radiomic features performed by two independent radiologists showed very good results for the absolute majority of indicators. This result indicates that the manual method of segmentation in the SliсerRadiomics application in the specified values of the adipose tissue attenuation may be employed by any operator. Thus, the choice in favor of the native image analysis is, in our opinion, sufficiently grounded.

In the assessment of the efficacy, the radiomic PAAT phenotype in the intergroup comparison had only 3 statistically significantly different features from the GLSZ matrix. This matrix is an example of the highest order matrices, which are suitable for the evaluation of non-periodic and non-uniform textures. It is used for quantitative description of grey level zones in the image determined by the number of connected pixels with equal intensity [33, 34]*^.^* The matrix contains 16 texture features each reflecting to some degree the uniformity and coarseness of the object texture. The Size Zone Non Uniformity feature and its normalized version, Size Zone Non Uniformity Normalized, quantify variability of the size zone volumes across the entire image, and the lower the value, the more homogeneous the size zone volumes are in the image. The Zone Entropy feature is a sort of an “antipode” to the Size Zone Non Uniformity and Size Zone Non Uniformity Normalized, and its large values indicate the heterogeneity of the texture in a random distribution of the zones and gray levels [32]. Without the histological analysis of the adipose tissue specimens, it is difficult to consider these factors in terms of pathomorphology. However, we believe that changes in these features in patients with postablation recurrence may be caused by the changes in the structure of the adipose tissue at the molecular level and characterize it as morphologically heterogeneous due to hypertrophy and hyperplasia of adipocytes. Of the given statistically significant textural features in the multifactorial logistic regression analysis, only one PAAT radiomic biomarker, Size Zone Non Uniformity Normalized, was an independent predictor of AF recurrence post ablation. Taking into account the correlation of Size Zone Non Uniformity, Size Zone Non Uniformity Normalized, and Zone Entropy with the left atrial diameter, the obtained results confirm again the mechanostructural type of action of the morphologically changed adipose tissue on the myocardium of the left atrium with the following fibrous remodeling.

### Study limitations

The study has several limitations. First, a single-center prospective cohort study of a small sample does not exclude the influence of systematic selection error and does not allow one to perform an adequate statistical analysis between the groups of patients with different AF forms. Second, due to a small sample size, we did not use advanced algorithms of machine learning and artificial intelligence. Third, the results of the texture analysis should be supported by the data on the level of biochemical markers of inflammation, such as hs-CRP (high-sensitivity C-reactive protein) and interleukin 6, comparative analysis with the results of intracardiac electrophysiological testing, as well as data of histological EAT investigation.

## Conclusion

A radiomic analysis of medical images is directed to the extraction of useful quantitative features, which may be the basis for building models for classification, prediction of the disease course, and response to treatment. Despite the fact that radiomic phenotype of the periatrial adipose tissue in patents with late postablation recurrence of atrial fibrillation is not pronounced, the application of this approach may be considered as a perspective non-invasive method of predicting poor outcome of catheter treatment, which opens the possibility for planning and correcting the tactics of patient management after the intervention.
